# Ribociclib Inhibits P-gp-Mediated Multidrug Resistance in Human Epidermoid Carcinoma Cells

**DOI:** 10.3389/fphar.2022.867128

**Published:** 2022-04-01

**Authors:** Lei Zhang, Biwei Ye, Yunfeng Lin, Yi-Dong Li, Jing-Quan Wang, Zhuo Chen, Feng-Feng Ping, Zhe-Sheng Chen

**Affiliations:** ^1^ Department of Pharmaceutical Sciences, College of Pharmacy and Health Sciences, St. John’s University, Queens, NY, United States; ^2^ State Key Laboratory of Structural Chemistry, Fujian Institute of Research on the Structure of Matter, Chinese Academy of Sciences, Fuzhou, China; ^3^ University of Chinese Academy of Sciences, Beijing, China; ^4^ Fujian Agriculture and Forestry University, Fuzhou, China; ^5^ Department of Reproductive Medicine, Wuxi People’s Hospital Affiliated to Nanjing Medical University, Wuxi, China

**Keywords:** cancer, ribociclib-LEE011, multidrug resistance (MDR), cyclin dependent kinases, CDKs 4 and 6, P-glycoprotein (ABCB1 protein)

## Abstract

The efficacy of cancer chemotherapy can be attenuated or abrogated by multidrug resistance (MDR) in cancer cells. In this study, we determined the effect of the CDK4/6 inhibitor, ribociclib (or LEE011), on P-glycoprotein (P-gp)-mediated MDR in the human epidermoid carcinoma MDR cell line, KB-C2, which is widely used for studying P-gp-mediated MDR in cancers. The incubation of KB-C2 cells with ribociclib (3–9 µM) increased the efficacy of colchicine, a substrate for P-gp. The cell expression of P-gp was down-regulated at both translation and transcription levels. Furthermore, ribociclib produced a 3.5-fold increase in the basal activity of P-gp ATPase, and the concentration required to increase basal activity by 50% (EC_50_) was 0.04 μM. Docking studies indicated that ribociclib interacted with the drug-substrate binding site of P-gp. The short-term and long-term intracellular accumulation of doxorubicin greatly increased in the KB-C2 cells co-cultured with ribociclib, indicating ribociclib inhibited the drug efflux activity of P-gp. The results of our study indicate that LEE011 may be a potential agent for combined therapy of the cancers with P-gp mediated MDR.

## Introduction

The cyclin-dependent kinases (CDKs) are members of serine-threonine kinases that regulate the cell cycle and affect cell proliferation and apoptosis (Braun et al., 1998; Diani et al., 2009; Szwarcwort-Cohen et al., 2009; Chung and Bunz, 2010; Hirai et al., 2010; Li et al., 2010; Duan et al., 2015; Rainey et al., 2017; Czudor et al., 2018; Menzl et al., 2019; Liang et al., 2020; Loyer and Trembley, 2020; Robert et al., 2020). They bind to their corresponding cyclin, forming a cyclin-CDK complex, where CDKs catalyze the phosphorylation of certain serine and threonine residues in target proteins, leading to regulation of gene transcription and cell division (Rank et al., 2000). The downregulation of CDK4/6 promotes cellular apoptosis, suppresses tumor proliferation, migration, and invasion (Liu et al., 2014; Guo et al., 2020; Hu et al., 2020). Currently, the CDK4/6 inhibitors, ribociclib, palbociclib and abemaciclib, have been approved by the U.S. Food and Drug Administration (FDA) for the treatment of breast cancer that is hormone receptor-positive and human epidermal growth factor receptor 2-negative, advanced or metastatic (Kwapisz, 2017; Tripathy et al., 2017) In cell-free assays, all three compounds are potent inhibitors of CDK4 and CDK6 (IC_50_ values from 2 to 39 nM) and ribociclib and abemaciclib inhibit CDK9 (IC_50_ of 1.51 μM and 57 nM, respectively) (Tripathy et al., 2017). Ribociclib induces the dephosphorylation of cyclin Rb, producing G_1_ phase cell cycle arrest (Aristizabal Prada et al., 2018; Tai et al., 2019). In addition, ribociclib can 1) overcome the resistance of OML1-R cancer cells to radiation (Tai et al., 2019) and 2) increase the anti-tumor efficacy of 5-fluorouracil or everolimus by downregulating the activity of the PI3K-Akt-mTOR and Ras-Raf-MEK-ERK pathways (Aristizabal Prada et al., 2018). Currently, the mechanisms involved in the reversal of MDR cancer by CDK4/6 inhibitors remains to be elucidated (Aristizabal Prada et al., 2018).

P-glycoprotein (P-gp/ABCB1) is an ATP-binding cassette (ABC) transporter that extrudes various anticancer drugs (e.g., paclitaxel, etoposide, vincristine, and doxorubicin) from cancer cells, as well as various endogenous molecules (Kathawala et al., 2015). The structure of P-gp consists of two symmetrical transmembrane domains (TMDs) formed by a group of helixes and two cytoplasmic nucleotide-binding domains (NBDs) in the form of a dimer occluding two ATPs, where the hydrolysis of ATP catalyzes the transport of P-gp substrates out of the cell (Kim and Chen, 2018). The overexpression of P-gp by various cancers can produce multidrug resistance (MDR), defined as the resistance to anticancer drugs that have distinct structures and differing mechanisms of action (Kathawala et al., 2015).

It has been reported that P-gp can significantly decrease the levels of ribociclib in the brain, suggesting that its efficacy may be limited by cancer cells that overexpress P-gp (Martinez-Chavez et al., 2019). Recently, it has been reported that ribociclib reverses the resistance to daunorubicin mediated by P-gp in acute myeloid leukemia cells by interacting with P-gp and inhibiting its efflux activity (Sorf et al., 2020). However, it remains to be ascertained if ribociclib reverses P-gp mediated MDR in solid tumor cells. Therefore, in this study, we determined the effect of ribociclib on P-gp-mediated MDR in cancer, by inhibiting the expression and the drug efflux activity of P-gp in the human epidermoid carcinoma MDR cell line, KB-C2. Our results indicated that ribociclib has effects on the ATPase activity of P-gp and, through direct interaction with P-gp, attenuates the activity of P-gp to extrude its substrate drugs, like colchicine and doxorubicin, further enhances the anticancer therapy efficacy of these drugs.

## Materials and Methods

### Cells, Plasmids, and Chemicals

The human epidermoid carcinoma cell line, KB-3-1, was used as the parental cell line. KB-C2, an MDR cell line that is resistant to colchicine due to the overexpression of P-gp, was created by exposing KB-3-1 cells to increasing concentrations of colchicine over a period of at least 2 months (Akiyama et al., 1985; Yoshimura et al., 1989). Both cell lines were kindly provided by Dr Shinichi Akiyama (Kagoshima University, Kagoshima, Japan). KB-3-1 and KB-C2 cells and their CDK4- and CDK6-deleted sublines were cultivated with DMEM supplemented with 10% FBS and 1% penicillin/streptomycin in a humidified incubator containing 5% CO_2_ at 37°C. CRISPR/Cas9 all-in-one plasmids, encoding single guide RNA (SgRNA) and Cas9, were purchased from GeneCopoeia Inc (Rockville, MD). Because KB-C2 cells are widely used for studying P-gp-mediated MDR in cancers, KB-C2 cells were used to determine the reversal of P-gp-mediated MDR.

Ribociclib was kindly provided by ChemieTek (Indianapolis, IN). Paclitaxel, colchicine, and doxorubicin were purchased form Sigma Chemical Co. (St. Louis, MO). Mouse anti-P-gp, HRP ligated or fluorescent secondary rabbit or goat-anti mouse antibodies, were purchased from Invitrogen, Thermo Fisher (Carlsbad, CA). Mouse-anti-CDK4 and CDK6 antibodies were purchased from R&D Systems (Minneapolis, MN). All other reagents were purchased from VWR International (West Chester, PA).

### Determination of Cell Viability: MTT Assay

Exponentially growing cells were seeded into 96 well plates at 5 × 10^3^ cells/well. These experiments were conducted in triplicate. After 72 h of incubation, 20 μl of MTT (5 mg/ml) was added to each well. After incubation for an additional 4 h, the medium containing MTT was discarded and replaced with 150 μl of DMSO. The plates were gently shaken until the dark blue-purple crystal were completely dissolved in DMSO. The absorbance was measured at a wavelength of 490 nm, using an ELx 800 Universal Microplate Reader (Bio-Tek, Inc. Winooski, VT). The relative survival rate (%) for the cells was analyzed using the SPSS 20 program (SPSS Inc., Chicago, IL) and the survival rate - drug concentration curves were generated using Origin 9.0 software (OriginLab corporation, Northampton, MA). The concentration of drug required to inhibit cell viability by 50% (IC_50_ value) was determined using Origin 9.0 software.

### Determination of the Efficacy of Ribociclib to Reverse MDR in Human Epidermoid Carcinoma KB-C2 Cancer Cells

Cells were seeded into 96-well plates (5 × 10^3^ cells per well) and cultured overnight. The cells were incubated with ribociclib (0, 0.3, 1 and 3 µM) for 1 or 2 h, followed by incubation with gradient concentrations of colchicine and paclitaxel, which are substrates for the P-gp transporter. The IC_50_ values of the anti-cancer drugs were determined using the MTT assay as described above.

### Western Blot Assay

Parental KB-3-1 cells and MDR KB-C2 cells were incubated with 9 μM of ribociclib for 2 h and co-cultured with paclitaxel or colchicine for 24–72 h. Western blot and immune-fluorescence (IF) assays were conducted to determine the effect of ribociclib on the expression of the P-gp protein. For the Western blot assay, the cells were lysed with SDS lysate reagent and separated on a gradient polyacrylamide gel (4%–20%, containing 0.1% SDS). The proteins on the SDS-PAGE gel were transferred to a PVDF membrane. After blocking with 5% milk, the membrane was washed with TBST buffer (150 mM NaCl, 10 mM Tris pH 8.0, 0.1% v/v Tween20) three times, incubated with mouse anti-P-gp antibody at 4°C for 2 h, adequately washed with TBST, and incubated with goat anti-mouse IgG-HRP (horseradish peroxidase) at RT for 2 h. The membrane was then washed with TBST four times and exposed to the SignalFire™ ECL Reagent developing reagent (Cell Singling Technology, Danvers, MA), and the results were quantified using an AI600 RGB GEL Imaging System (GE, Fairfield, CT) set for the chemiluminescence mode.

### RT-Quantitative-PCR Analysis of *ABCB1* Transcription

RT-PCR of *ABCB1* mRNA level was performed to investigate the influence of ribociclib on the ABCB1 expression on the transcription level. Ribociclib (9 µM) showing the effect of reversing ABCB1 mediated MDR in cancer cells was added to the cell culture medium. The cells cultured without ribociclib were set as control. After 48 h of cell culture, the cells were sampled for mRNA extraction and RT-q-PCR (QuantStudio™ 5 Real-Time PCR System, ThermoFisher Scientific, CA). *ABCB1* and *GAPDH* were the objective and internal reference genes, respectively. Primers for amplification of *ABCB1* gene were: GAAAGTGAAAAGGTTG (Forward), and CTGGCGCTTTGTTCCA (Reverse). Primers for amplification of *GAPDH* gene were ATTGACCTCAACTACA (Forward), and AGAGATGATGACCCTT (Reverse). Expression deviation was calculated according to the formular: Ratio _Sample/Control_ = 2^−ΔΔCT^, where C_T_ value was automatically calculated by QuantStudio™ Design and Analysis Software v1.3.1 according to the ΔRn-Cycle curve.

### ATPase Activity Assay

The ATPase activity of P-gp was determined using the PREDEASY ATPase Kits (SOLVO Biotechnology, Szeged, Hungary), according to the manufacturer’s protocol (Ambudkar, 1998). Briefly, the membranes (20 μg) were incubated in assay buffer (50 mM of MES at pH 6.8, 50 mM of KCl, 5 mM of sodium azide, 2 mM of EGTA, 2 mM of DTT, 1 mM of ouabain and 10 mM of MgCl_2_). The membrane vesicles from Sf9 cells were provided by the manufacturer, expressing high levels of human ABCB1, were incubated with ribociclib (0.05, 0.1, 0.2, 0.4, 0.6, 0.8, 1, 5, 10 and 20 µM) for 3 min. ATP hydrolysis was initiated by adding 5 mM of Mg-ATP and the reaction was terminated using a 5% SDS solution. Subsequently, the light absorption was measured at 800 nm using a Bio-Rad SmartSpec 30,000 UV/Vis spectrophotometer (Bio-Rad Laboratories, Hercules, CA).

### Efflux of Doxorubicin by Accumulation Assay

Because doxorubicin is a fluorescent substrate that can be extruded from cancer cells by the P-gp transporter (Mi et al., 2010), its intracellular accumulation was used to determine if ribociclib can directly inhibit the efflux function of the P-gp transporter, thereby increasing the accumulation of doxorubicin. The parental and MDR cells were incubated with 9 µM of ribociclib for 1 h. Doxorubicin (0.2 µM) was added and co-incubated with the cells for 2 h. This incubation period and the relatively low concentration of doxorubicin were used to maintain cell viability. The cells were gently washed with PBS buffer three times and lysed using SDS lysate reagent (50 mM, pH 8.0 Tris, 1% SDS, 2 mM of sodium pyrophosphate, 25 mM of *β*-glycerophosphate, 1 mM of EDTA, 1 mM of Na_3_VO_4_ and 0.5 μg/ml of leupeptin). The fluorescence intensity, an indicator of doxorubicin accumulation, was determined using a SynergyTM 4 Multi-Mode Microplate Reader (Bio Tek Instruments, Inc. VT, Excitation/Emission: 450/550 nm).

For the long-term evaluation of the efflux of doxorubicin, cells were seeded at 3 × 10^3^ cells per well, cultured for 8 h, and incubated with 9 μM of ribociclib and doxorubicin (1 µM for the MDR KB-C2 cells and 0.1 µM for drug-sensitive KB-3-1 cells to maintain MDR and cell viability above 60%) for 72 h. Because the membrane of the dead or severely apoptotic cells was damaged, which could lead to the inaccurate quantification of the intercellular doxorubicin levels, the distribution of doxorubicin was determined in triplicate, using fluorescent microscopy.

### Docking Analysis of the Interaction of Ribociclib With CDK4, CDK6 and the P-gp Transporter

We first determined the transcripts for the P-gp and CDK4/6 proteins in the KB-C2 cell line. According to the open reading frame (ORF) sequences detected in the major transcripts of P-gp in KB-C2 cell line, the structure was achieved by structure modelling using a Swiss model platform (https://swissmodel.expasy.org/). The interactions between ribociclib and P-gp, CDK4 or CDK6 were calculated using HEX 8.0 software (LORIA/Inria Nancy Research Centre in Nancy, France), based on the most stable calculated structures of the complexes by molecular docking (Kaczor et al., 2013). PyMOL (version 1.8. x) was used to analyze the data and determine the most stable complex structures, binding positions (such as residues or chemical groups) and interactions.

## Results

### Ribociclib Significantly Increases the Efficacy of Colchicine in KB-C2 Cancer Cells Overexpressing the P-gp Transporter

**FIGURE 1:**
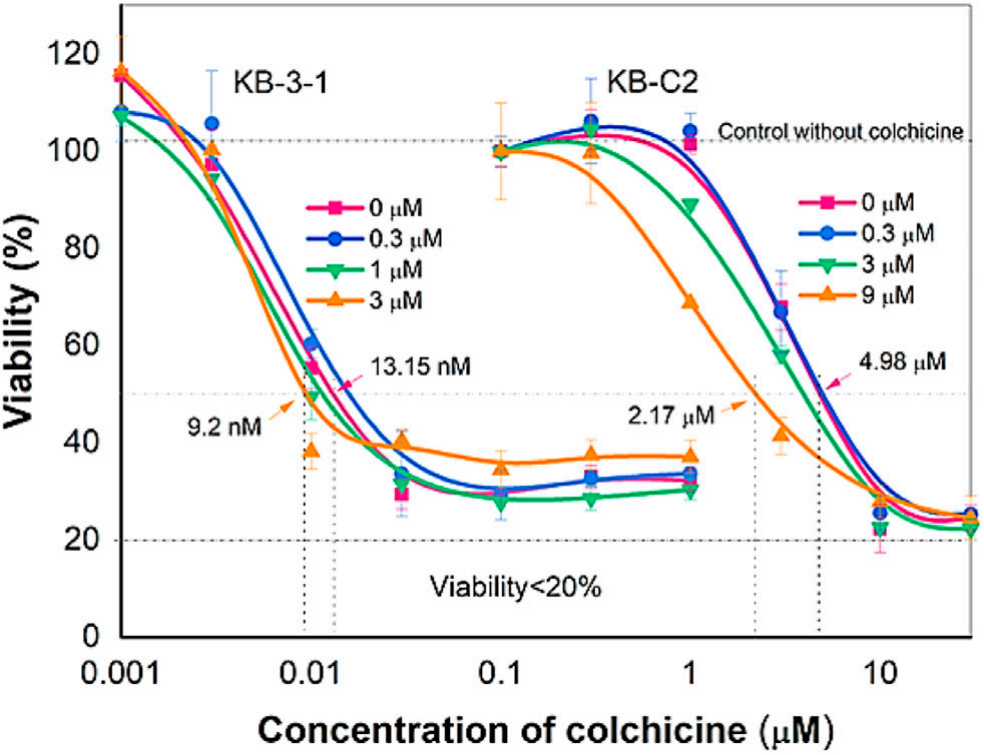
Reversal effect of ribociclib on MDR in KB-C2 cell line. Ribociclib reverses MDR in KB-C2 cells but does not significantly affect the efficacy of colchicine in the parental KB-3-1 cells. Ribociclib at 9 µM significantly decreased the IC_50_ of colchicine in KB-C2 cancer cells (*p* < 0.05).

We conducted experiments to determine if ribociclib could increase the efficacy of colchicine (i.e., reversing resistance to colchicine) in MDR KB-C2 cancer cells. As previously reported, colchicine potently decreased (IC_50_ = 13.15 nM) the viability of the colchicine-sensitive KB-3-1 cancer cells (Figure 1). In contrast, the IC_50_ value for colchicine was 4.98 µM in the colchicine-resistant KB-C2 cancer cells, indicating these cells were almost three orders of magnitude more resistant to colchicine compared to the parental KB-3-1 cancer cells (Figure 1).

Ribociclib non-significantly decreased the IC_50_ value of colchicine in the KB-3-1 cancer cells, whereas in the colchicine-resistant KB-C2 cancer cells, 9 µM of ribociclib significantly decreased the IC_50_ of colchicine in KB-C2 cancer cells (Figure 1). These results suggested that the resistance of KB-C2 cancer cells to colchicine, which was mediated by the overexpression of the P-gp transporter^36^, could be partially attenuated by ribociclib (Figure 1).

### Ribociclib Significantly Down-Regulates the Expression of the P-gp Transporter

**FIGURE 2:**
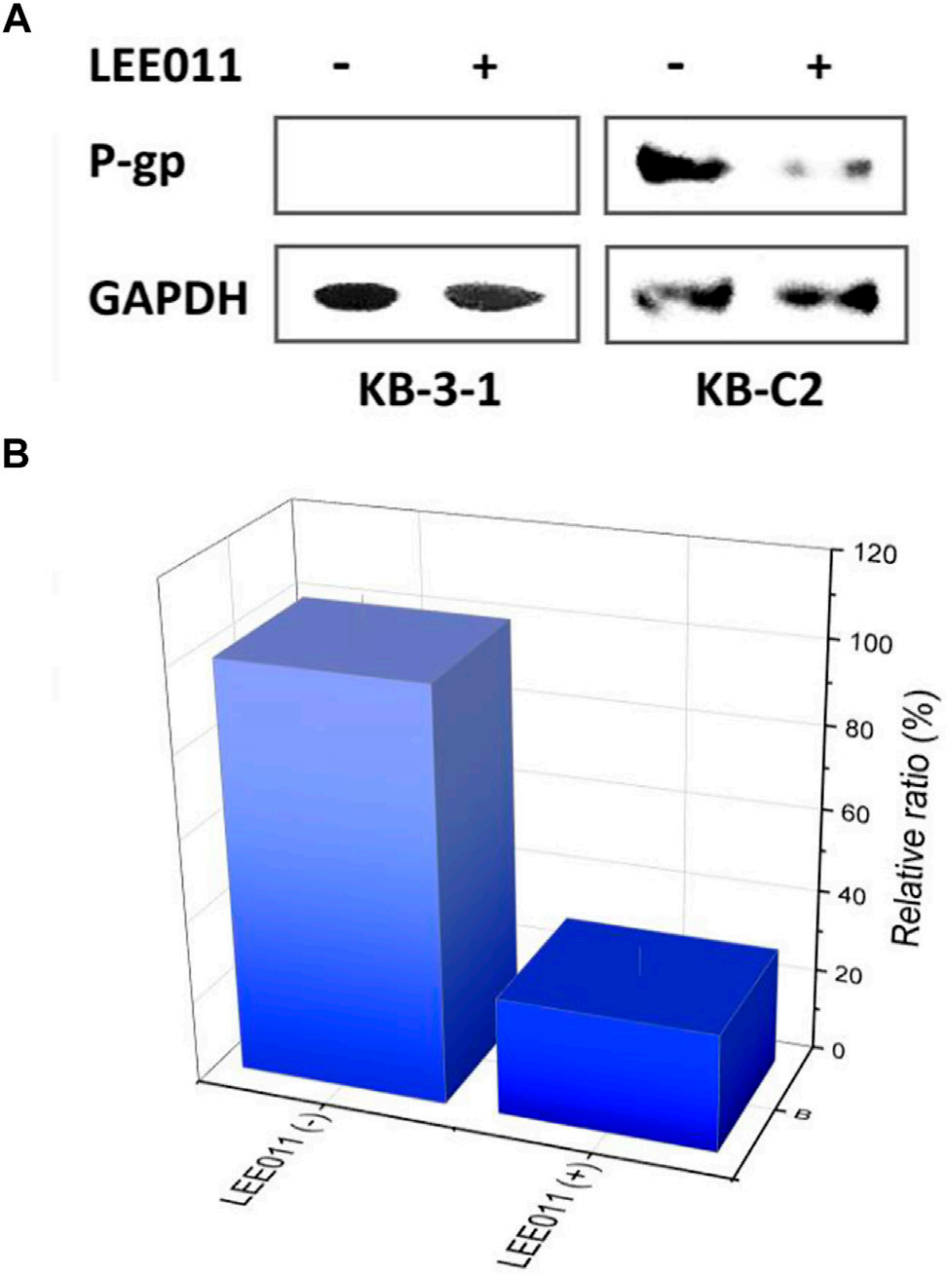
ABCB1 expression deviation between the robociclib/LEE011 treated KB-C2 cells and the robociclib non-treated KB-C2 cells. **(A)** Western blot indicated that P-gp expression was significantly downregulated by incubating the cells with 9 µM of ribociclib for 48 h **(B)** *ABCB1* mRNA level was analyzed by RT-q-PCR, using *GAPDH* mRNA as the inner reference. The cells without robociclib treatment were set as control.

It is possible that ribociclib increases the efficacy of colchicine in KB-C2 cancer cells by affecting the expression of the P-gp protein. Therefore, we used Western blotting to determine the effect of ribociclib on P-gp expression. Our results indicated the incubation of KB-C2 cancer cells with 9 µM (a non-toxic concentration) of ribociclib for 72 h produced a significant decrease in the expression of P-gp protein levels compared to cells incubated with vehicle (Figure 2A). The remaining P-gp transporters expressed by the KB-C2 cells most likely mediated the lowered drug resistance produced by P-gp (Figure 2A). In contrast, P-gp protein expression was not significantly altered in KB-3-1 cancer cells incubated with 9 µM of ribociclib compared to cells incubated with vehicle (Figure 2A).

Treatment with ribociclib remarkably downregulated *ABCB1* transcription in the KB-C2 cells (Figure 2B). Approximately 5.6% of the *ABCB1* mRNA amounts was detected in the cells treated with ribociclib (9 µM), as compared with that detected in the cells without ribociclib treatment. This conclusion was coherent with the that supported by Western blot (Figure 2B). This phenomenom implied that the ribociclib down regulated P-gp at both the translational and transcriptional levels.

### Ribociclib’s Interaction With a Human Homology Model of the P-gp Transporter

**FIGURE 3:**
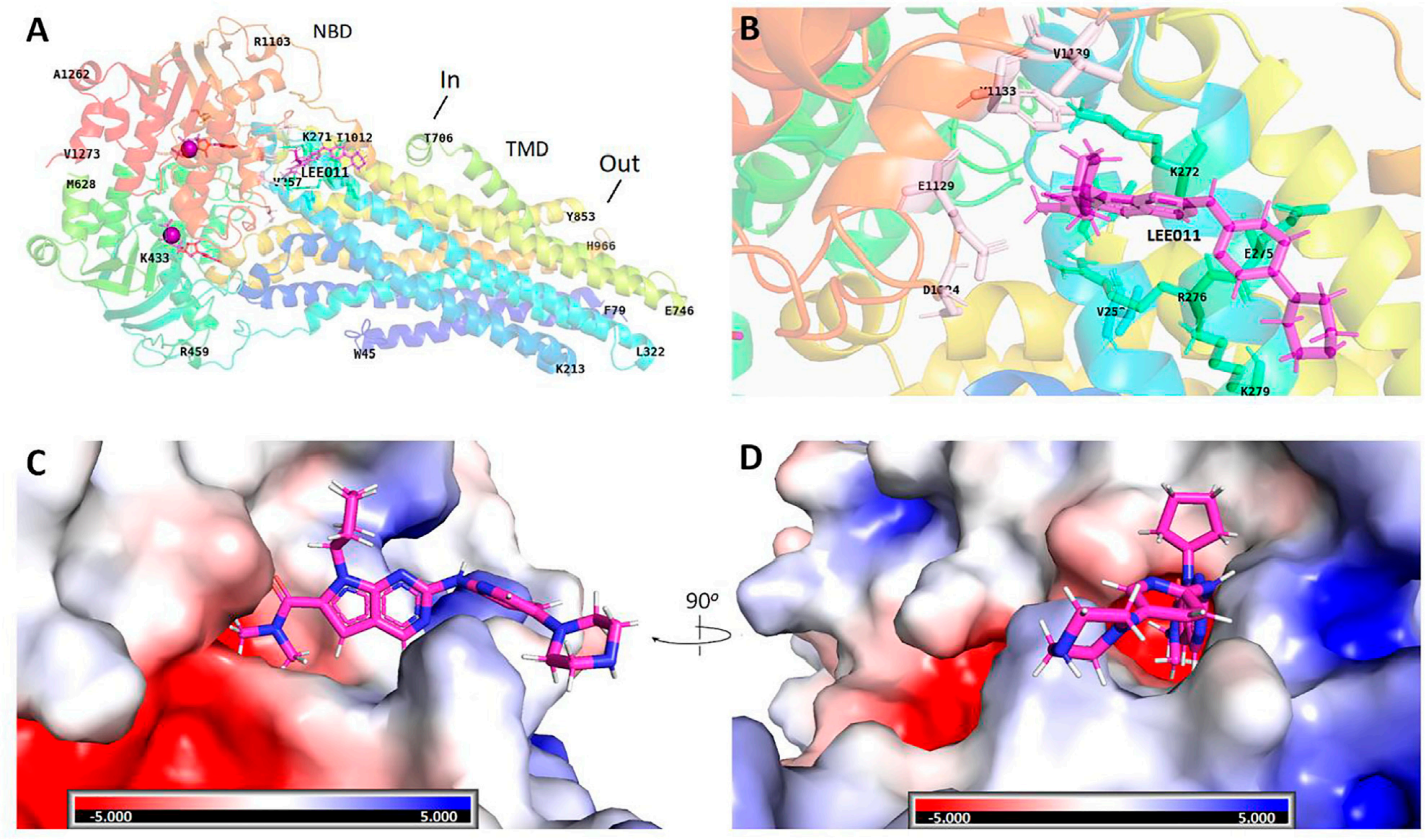
Structural basis for the interaction of ribociclib with P-gp. Docking analysis of the 3-dimentional structure of the ribociclib-P-gp complex were performed using HEX 8.0 software. **(A)** Ribociclib interacted with the NBD domain near the interface at the inner side. **(B)** The magnified region showing the amino groups that interact with ribociclib **(C, D)** Spatial structure and charge distributions of the site that binds ribociclib.

**FIGURE 4:**
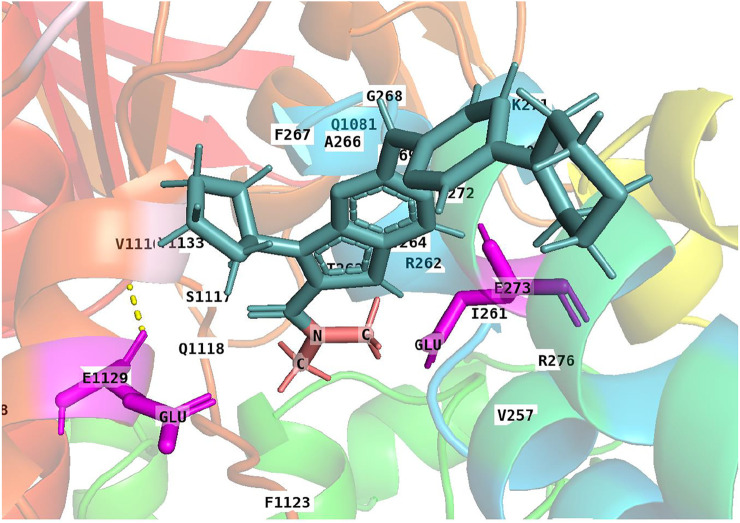
A 3-D structural model showing the electrostatic interaction between ribociclib and P-gp. Under physiological conditions, the N,N-dimethylamide cluster is positively charged and may bind to a cavity containing E273 and E1129 with negative charges due to the dissociation of hydrogen protons from the carboxyl group at neutral or higher pH values.

Although ribociclib showed decent inhibition activity when it binds CDK4 or CDK6 (Supplementary Figures S1–S4), we also performed docking analysis experiments to determine if ribociclib interacted with the P-gp transporter and if so, what chemical interactions were involved. Our results indicated that a strong interaction between P-gp and ribociclib existed. Docking studies indicated that ribociclib interacted with the drug-substrate binding site of P-gp, and had a Docking Score/Etotal (Eforce + Eshape, Kalaiselvi et al., 2015) of - 271.06. The molecular modeling indicated electrostatic interactions between N,N-dimethylamide cluster (positively charged with a proton at physiological conditions) in ribociclib and E273 and E1129 (with negative charges), in a trough-like structure between TMDs and NBDs in P-gp, which is adjacent to the interphase of the inner membrane (Figure 3, Figure 4). Since ribociclib was estimated to bind in a non-representative, drug-substrate pocket of P-gp, it was unknown as to whether this interaction results in a change in the efflux function of P-gp, which is frequently associated with a change in the ATPase activity of P-gp and the intracellular accumulation of antitumor drugs (Zhang et al., 2020). Therefore, we conducted experiments to determine if ribociclib affected ATPase activity.

### Ribociclib Increases the ATPase Activity of the P-gp Transporter

Although ribociclib has been reported to be highly efficacious in inhibiting CDK4/6 (Kwapisz, 2017; Tripathy et al., 2017), it remains to be elucidated whether ribociclib interacts with P-gp. Therefore, we conducted experiments to determine if ribociclib 1) interacts directly with P-gp and alters the efflux activity and 2) alters ATPase activity of human P-gp in the membrane vehicles.

**FIGURE 5:**
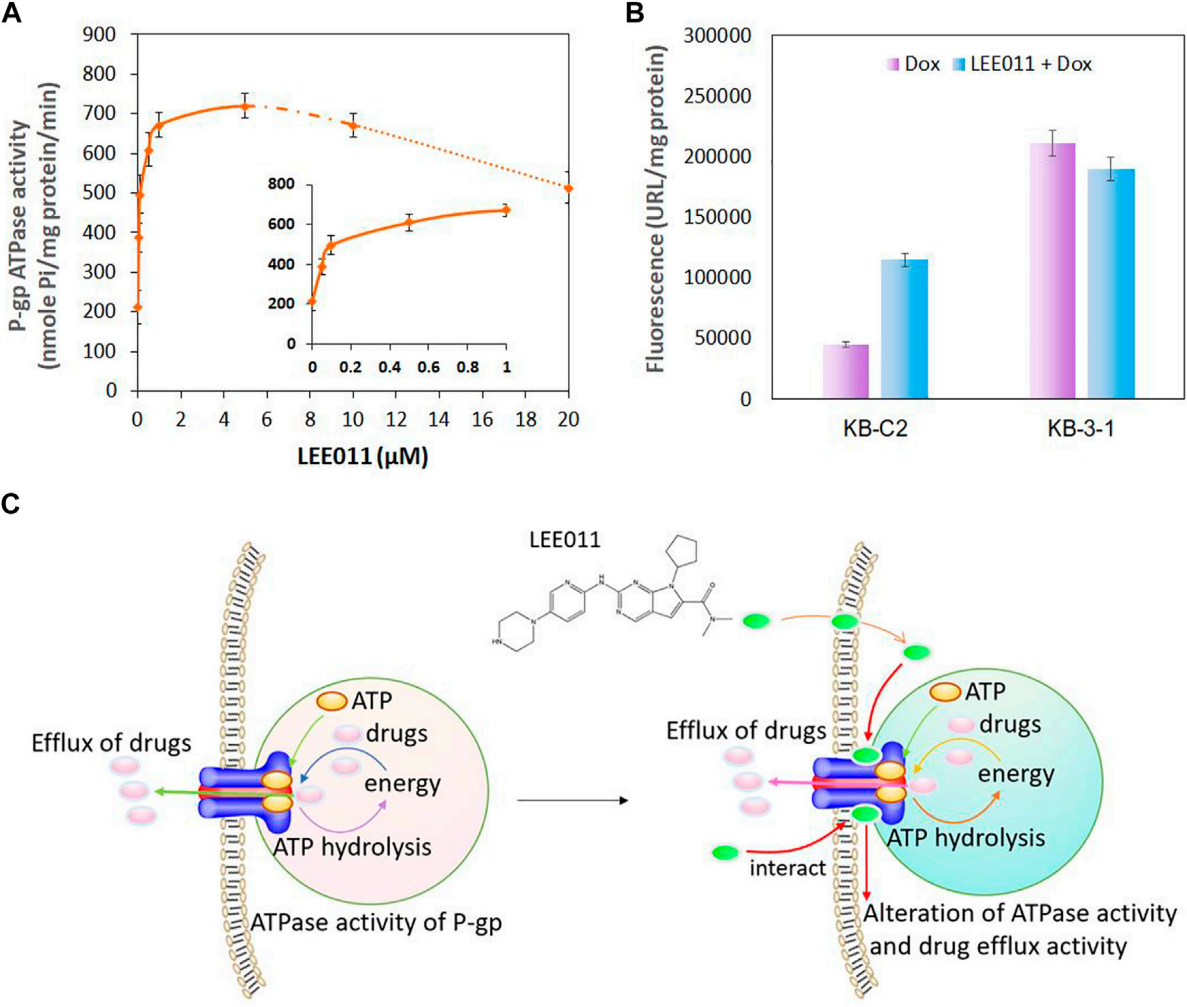
The interaction between ribociclib and the P-gp transporter alters the ATPase activity of P-gp and drug efflux activity in KB-C2 cells. **(A)** ATPase activity of P-gp following incubation with ribociclib. **(B)** A comparison of the drug accumulation in MDR KB-C2 cells over-expressing P-gp and the drug sensitive KB-3-1 cells in the presence or absence of ribociclib. The cells were co-cultured with ribociclib (9 µM) and Dox (0.2 µM) for 2 h. The level of Dox fluorescence was measured at 550 nm. **(C)** A Scheme showing that ribociclib alters ATPase activity and drug efflux by interacting with the P-gp transporter.

Studies have shown that P-gp transporter hydrolyzes ATP, which is involved in drug efflux (Kim and Chen, 2018) and ATPase activity can be stimulated or inhibited by various P-gp substrates (Feng et al., 2020; Wu et al., 2020). It has been postulated that the stimulation of the P-gp ATPase activity by an experimental compound suggests that it is interacting with the transporter at the drug-substrate binding site (Zhang et al., 2020). Therefore, we determined the effect of various concentrations of ribociclib on P-gp ATPase activity. The incubation of membrane vesicles from sf9 insect cells (which express high levels of P-gp) with ribociclib (0.05–20 µM) produced a maximal increase of 3.5-fold in the basal activity of the P-gp transporter ATPase and the EC_50_ value was 0.04 μM (Figure 5A). The stimulation of P-gp transporter ATPase activity by ribociclib suggests that it may interact with the transporter at the drug-substrate binding site, although this remains to be definitively proven.

### The Effect of Ribociclib on the Intracellular Accumulation of Doxorubicin in KB-3-1 and KB-C2 Cancer Cells

It is possible that ribociclib reverses the drug resistance of KB-C2 cancer cells by inhibiting the efflux function of P-gp. Therefore, we determined the effect of ribociclib (using a 2 h incubation period) on the intracellular accumulation on doxorubicin, a substrate for the P-gp transporter, in KB-C2 cancer cells. As previously reported (Zhang et al., 2020), the accumulation of doxorubicin was significantly greater in the parental cell line, KB-3-1, compared to MDR KB-C2 cells, which overexpress the P-gp transporter (Figure 5B) (Akiyama et al., 1985; Yoshimura et al., 1989). Doxorubicin accumulation was significantly increased in KB-C2 cancer cells incubated with 9 µM of ribociclib compared to cells incubated with vehicle (Figure 5B). In contrast, doxorubicin accumulation in the parental KB-3-1 cells, which do not overexpress the P-gp transporter, was not significantly altered by 9 µM of ribociclib. The relationship between the interaction of ribociclib with the P-gp transporter and its inhibition of drug efflux and increase in ATPase activity is summarized in Figure 5C.

These results indicated that ribociclib increases the ATPase activity of P-gp and inhibits the drug-efflux function of P-gp, suggesting that ribociclib can interact with P-gp directly, which may contribute to the reversal of P-gp-mediated MDR in KB-C-2 cells.

### The Effect of the Incubation of KB-3-1 and KB-C2 Cancer Cells With Ribociclib for 72 h on the Intracellular Accumulation of the P-gp Transporter Substrate, Doxorubicin

**FIGURE 6:**
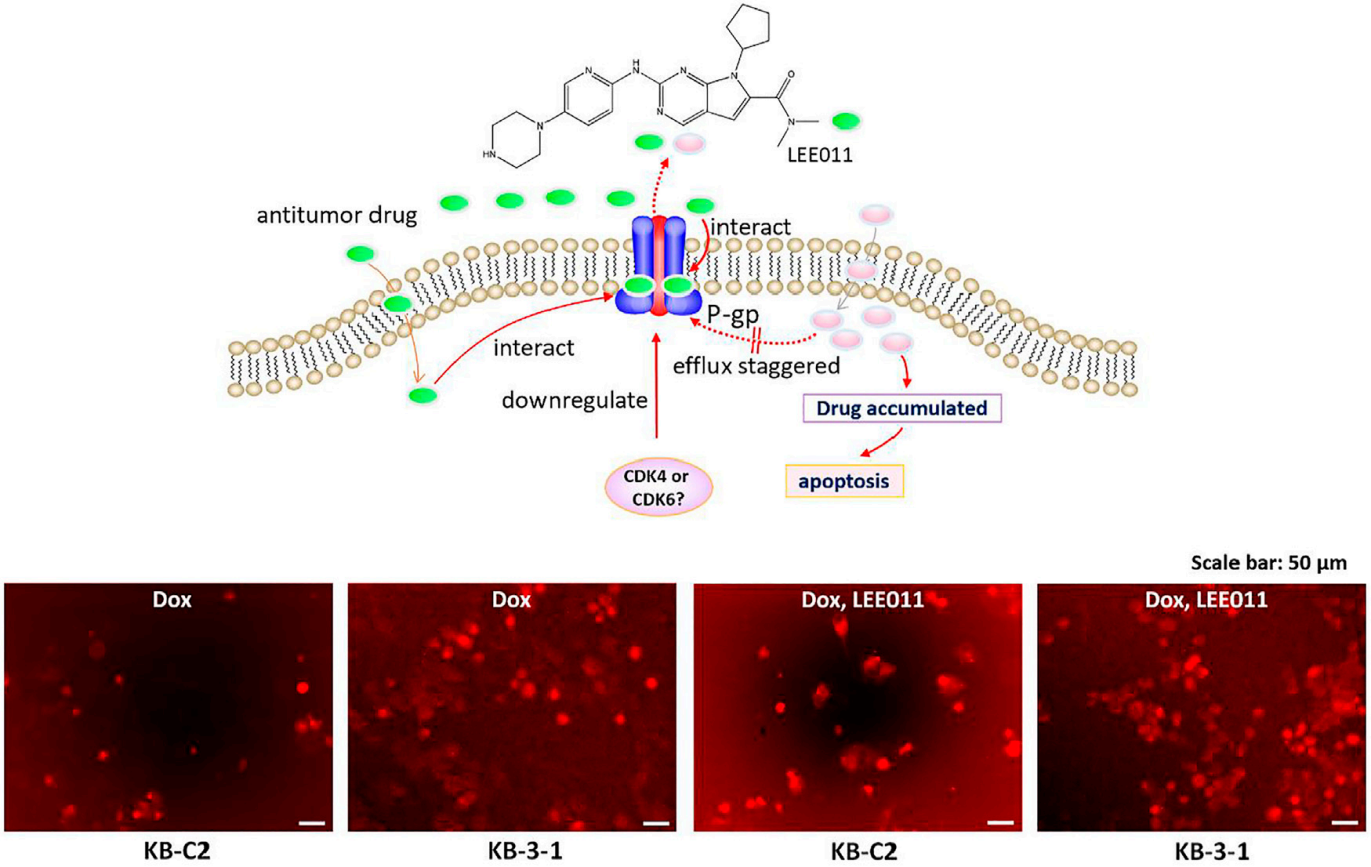
Ribociclib significantly increases the accumulation of doxorubicin (Dox) in KB-C2 cells after 72 h of incubation, producing apoptosis or necrosis. KB-C2 and parental KB-3-1 cells were co-cultured with doxorubicin (1 µM for KB-C2 and 0.1 µM for KB-3-1) and ribociclib 1 (9 µM) for 72 h. The cells incubated with vehicle were used as controls. The fluorescence of Dox was imaged using fluorescent microscopy (set to the red fluorescent channel). The schematic figure shows that ribociclib increases the accumulation of Dox by 1) decreasing its efflux, which increases apoptosis and 2) downregulating the levels of P-gp protein expression which also increases the intracellular levels of Dox.

These studies were conducted to ascertain if the prolonged incubation (72 h) of KB-3-1 and KB-C2 cancer cells with ribociclib would inhibit the efflux of doxorubicin. The incubation of the parental KB-3-1 cells with 9 µM of ribociclib for 72 h did not significantly alter the intracellular accumulation of doxorubicin compared to cells incubated with vehicle (Figure 6). We concluded that the presence of ribociclib had no effect on the accumulation of DOX within KB-3-1 cells, which did not express P-gp and could not extrude DOX efficiently (Zhang et al., 2022). In contrast, doxorubicin accumulation was significantly increased in the KB-C2 cells incubated with 9 µM of ribociclib compared to cells incubated with vehicle (Figure 6). Thus, the accumulation of doxorubicin in the KB-C2 cells that overexpress the P-gp transporter is significantly increased after 2 or 72 h of incubation with 9 µM of ribociclib, suggesting that ribociclib increases the intracellular accumulation of doxorubicin by inhibiting the efflux function of P-gp in KB-C2 cells.

## Discussion

Cancers with multidrug resistance caused by overexpression of P-gp are one of the major causes for failure of chemotherapy. Seeking for inhibitors of P-gp is an applicable approach for improving the efficiency of MDR treatment. As previously reported, KB-C2 cancer cells, which overexpress P-gp, were highly resistant to colchicine, a substrate for P-gp (Yang et al., 2014). Ribociclib, at 9 μM, a concentration that did not affect cell viability, significantly decreased the IC_50_ value of colchicine (i.e., decreased drug resistance) in the KB-C2 cells, whereas it had no significant effect on the efficacy of colchicine in the parental KB-3-1 cells (Figure 1). To our knowledge, this is the first study to show that ribociclib decreases resistance of KB-C2 cancer cells to colchicine. Subsequently, we conducted studies to determine the mechanism(s) by which ribociclib reverses resistance to the P-gp substrates, colchicine and doxorubicin, in human epidermoid carcinoma KB-C2 cells, which have been frequently used to study P-gp-mediated MDR in cancers (Akiyama et al., 1985; Yoshimura et al., 1989). It is possible that ribociclib could reverse resistance by inhibiting the efflux function and/or the expression of the P-gp transporter, thereby increasing the intracellular concentration of substrate drugs. Our results indicated that, the intracellular accumulation of doxorubicin, a substrate of P-gp transporter, was significantly decreased in the drug resistant KB-C2 cancer cells compared to the KB-3-1 cancer cells. The incubation of KB-C2 cancer cells with 9 µM of ribociclib, for 2 or 72 h, produced a 2-fold increase in the accumulation of doxorubicin in KB-C2 cells compared to vehicle (Figure 5). However, ribociclib did not significantly alter the concentration of doxorubicin in KB-3-1 cancer cells, which do not overexpress the P-gp transporter. Previously, it has been reported that abemaciclib, a CDK4/6 inhibitor (Iriyama et al., 2018), increased the intracellular accumulation of doxorubicin by competitively inhibiting P-gp - or ABCG2-mediated drug efflux in cells overexpressing these transporters (Wu et al., 2017).

These data tentatively suggested that ribociclib can reverse resistance to P-gp substrates in KB-C2 cancer cells by suppressing the expression of P-gp. To further delineate the mechanism of action of ribociclib, we conducted docking study to determine the magnitude of the interaction of ribociclib with a human homology model of the P-gp transporter (Figure 3, Figure 4). The result showed that ribociclib significantly interacts with the P-gp transporter. In addition to the surface matching and van der Waals interactions, there is a strong electrostatic interaction between the N,N-dimethylamide cluster in ribociclib and the amino acids E273 and E1129 in P-gp protein. These results, in addition to the intracellular drug accumulation data, suggest that ribociclib interacts with the drug-substrate binding pocket of P-gp, further suggesting that it could be a substrate of P-gp that inhibits the binding of other P-gp substrates, such as colchicine and doxorubicin.

Ribociclib could reverse P-gp-mediated MDR by decreasing the expression of the P-gp transporter. Western blot and IF data indicated that the incubation of KB-C2 cells with 9 μM of ribociclib for 72 h significantly decreased the expression level of the P-gp protein compared to cells incubated with vehicle (Figure 1). In contrast to our results with ribociclib, it has been reported that abemaciclib, a CDK4/6 inhibitor, did not significantly alter the expression of the P-gp transporter in cancer cells overexpressing the P-gp transporter (Wu et al., 2017). The exact explanation for the differential effect of abemaciclib and ribociclib on the expression level of the P-gp remains to be determined. However, this could be due to differences in their intracellular accumulation and interaction with their targets. Overall, ribociclib decreases the resistance to colchicine and doxorubicin in KB-C2 cancer cells by 1) interacting with the P-gp protein and inhibiting the efflux function, thereby increasing the intracellular accumulation of these anticancer drugs and 2) decreasing the expression of the P-gp transporter, which decreases the number of transporters and thus, drug efflux.

Studies have reported that certain compounds or drugs can stimulate the ATPase activity of ABC transporters by binding to the substrate-drug binding site (Lee et al., 2019; Li et al., 2020). In this study, ribociclib produced a significant increase (3.5-fold) in the ATPase activity of the P-gp transporter (Figure 5A). This result suggests that ribociclib interacts with the substrate-drug binding site which could inhibit the binding of other P-gp transporter substrates, further inhibiting their efflux, thus increasing their intracellular levels. Similarly, voruciclib, a CDK4/6 inhibitor (Gupta et al., 2018; Gupta et al., 2018), significantly increased the ATPase activity of P-gp and inhibited the efflux of paclitaxel or mitoxantrone from human colorectal adenocarcinoma SW620/AD300 cells overexpressing P-gp and non-small cell lung cancer NCI-H460/MX20 cells overexpressing BCRP, respectively, thus reversing the MDR mediated by P-gp and BCRP, respectively (Gupta et al., 2018). However, the expression level of P-gp and BCRP was not significantly altered by the incubation of cells with 5 µM of voruciclib (Gupta et al., 2018). The difference between ribociclib and voruciclib on the expression level of P-gp could be due to their differential interaction with proteins that control the transcription of the P-gp protein, although this remains to be elucidated.

When the KB-C2 cells were cocultured with ribociclib at higher concentrations (exceeding 9 µM), reversal effects on MDR in KB-C2 cells increased. But the cell proliferation was also inhibited when using only ribiciclib (instead of colchicine) higher concentrations. This could be caused by the enhanced inhibition effect on CDK4/6 by a greater number of ribociclib molecules that had entered the cells in addition to their interaction with the membrane P-gp transporters. Thus, the functions of inhibiting cancer cell proliferation and reversing P-gp mediated MDR could synergize each other during combined chemotherapy based on ribociclib and P-gp substrate drugs.

By single gene knockout using CRISPR/Cas9 technique in human epidermoid carcinoma MDR cell line KB-C2, we recently revealed that CDK6-PI3K signaling axis is an efficient target for attenuating ABCB1/P-gp mediated MDR in cancer cells (Zhang et al., 2022). CDK6 knockout downregulated PI3K 110*α* and 110*β*, and PI3K 110*α*/110*β* deficiency in-return downregulated CDK6. CDK6-PI3K axis synergizes in regulating ABCB1 expression, which further strengthened the regulation of ABCB1 over single regulation by either CDK6 or PI3K 110*α*/110*β*.

It will be instructive if we know the mechanisms of the interaction between ribociclib and P-gp, which will benefit modification of the structure of ribociclib and improving its affinity to P-gp. Till now, no study about this aspect has been reported, however, we are still making efforts on its exploration, which is an undergoing project in our laboratories.

In our laboratories, we are currently performing modification and optimization of a serial of combined drug-systems that can reverse MDR in cancers and inhibit cancer cell growth as well. Based on these studies, our tasks in the next stage will contain animal study to testify the biocompatibility and tumor killing efficacy of these drug systems.

In conclusion, the results of this study indicated that in KB-C2 cells, ribociclib inhibited the efflux of the P-gp transporter substrates, doxorubicin and colchicine and decreased the expression of the P-gp transporter, resulting in the reversal of MDR. P-gp expression was downregulated by ribociclib. Furthermore, protein docking data reveals that ribociclib binds near the P-gp transporter drug-substrate binding site and it stimulates the basal activity of the P-gp ATPase. ATPase analysis and drug accumulation experiments further demonstrated that the activity of P-gp was inhibited by ribociclib. Thus, ribiciclib may be a promising inhibiter for the application in combination of anticancer therapies against solid tumor cells with P-gp mediated MDR.

## Data Availability

The original contributions presented in the study are included in the article/Supplementary Material, further inquiries can be directed to the corresponding authors.

## References

[B1] Akiyama S. Fojo A. Hanover J. A. Pastan I. Gottesman M. M. (1985). Isolation and Genetic Characterization of Human KB Cell Lines Resistant to Multiple Drugs. Somat Cel Mol Genet 11, 117–126. 10.1007/BF01534700 3856953

[B2] Ambudkar S. V. (1998). Drug-stimulatable ATPase Activity in Crude Membranes of Human MDR1-Transfected Mammalian Cells. Methods Enzymol. 292, 504–514. 10.1016/s0076-6879(98)92039-0 9711578

[B3] Aristizabal Prada E. T. Nölting S. Spoettl G. Maurer J. Auernhammer C. J. (2018). The Novel Cyclin-dependent Kinase 4/6 Inhibitor Ribociclib (LEE011) Alone and in Dual-Targeting Approaches Demonstrates Antitumoral Efficacy in Neuroendocrine Tumors *In Vitro* . Neuroendocrinology 106, 58–73. 10.1159/000463386 28226315

[B4] Braun K. Hölzl G. Pusch O. Hengstschläger M. (1998). Deregulated Expression of CDK2- or CDK3-Associated Kinase Activities Enhances C-Myc-Induced Apoptosis. DNA Cel Biol 17, 789–798. 10.1089/dna.1998.17.789 9778038

[B5] Chung J. H. Bunz F. (2010). Cdk2 Is Required for P53-independent G2/M Checkpoint Control. Plos Genet. 6, e1000863. 10.1371/journal.pgen.1000863 20195506 PMC2829054

[B6] Czudor Z. Balogh M. Bánhegyi P. Boros S. Breza N. Dobos J. (2018). Novel Compounds with Potent CDK9 Inhibitory Activity for the Treatment of Myeloma. Bioorg. Med. Chem. Lett. 28, 769–773. 10.1016/j.bmcl.2018.01.002 29329658

[B7] Diani L. Colombelli C. Nachimuthu B. T. Donnianni R. Plevani P. Muzi-Falconi M. (2009). Saccharomyces CDK1 Phosphorylates Rad53 Kinase in Metaphase, Influencing Cellular Morphogenesis. J. Biol. Chem. 284, 32627–32634. 10.1074/jbc.M109.048157 19801655 PMC2781677

[B8] Duan C. Liu Y. Lu L. Cai R. Xue H. Mao X. (2015). CDK14 Contributes to Reactive Gliosis via Interaction with Cyclin Y in Rat Model of Spinal Cord Injury. J. Mol. Neurosci. 57, 571–579. 10.1007/s12031-015-0639-x 26315607

[B9] Feng W. Zhang M. Wu Z. X. Wang J. Q. Dong X. D. Yang Y. (2020). Erdafitinib Antagonizes ABCB1-Mediated Multidrug Resistance in Cancer Cells. Front. Oncol. 10, 955. 10.3389/fonc.2020.00955 32670878 PMC7330633

[B10] Guo Z. Lv X. Jia H. (2020). MiR-186 Represses Progression of Renal Cell Cancer by Directly Targeting CDK6. Hum. Cel 33, 759–767. 10.1007/s13577-020-00357-3 32266659

[B11] Gupta P. Zhang Y. K. Zhang X. Y. Wang Y. J. Lu K. W. Hall T. (2018). Voruciclib, a Potent CDK4/6 Inhibitor, Antagonizes ABCB1 and ABCG2-Mediated Multi-Drug Resistance in Cancer Cells. Cell Physiol. Biochem. 45, 1515–1528. 10.1159/000487578 29486476

[B12] Hirai H. Shimomura T. Kobayashi M. Eguchi T. Taniguchi E. Fukasawa K. (2010). Biological Characterization of 2-Aminothiazole-Derived Cdk4/6 Selective Inhibitor *In Vitro* and *In Vivo* . Cell Cycle 9, 1590–1600. 10.4161/cc.9.8.11306 20372067

[B13] Hu Q. L. Xu Z. P. Lan Y. F. Li B. (2020). MiR-636 Represses Cell Survival by Targeting CDK6/Bcl-2 in Cervical Cancer. Kaohsiung J. Med. Sci. 36, 328–335. 10.1002/kjm2.12181 31889428 PMC11896350

[B14] Iriyama N. Hino H. Moriya S. Hiramoto M. Hatta Y. Takei M. (2018). The Cyclin-dependent Kinase 4/6 Inhibitor, Abemaciclib, Exerts Dose-dependent Cytostatic and Cytocidal Effects and Induces Autophagy in Multiple Myeloma Cells. Leuk. Lymphoma 59, 1439–1450. 10.1080/10428194.2017.1376741 28918692

[B15] Kaczor A. A. Selent J. Sanz F. Pastor M. (2013). Modeling Complexes of Transmembrane Proteins: Systematic Analysis of Protein; Protein Docking Tools. Mol. Inform. 32, 717–733. 10.1002/minf.201200150 27480064

[B16] Kalaiselvi M. Amsaveni R. Bhuvaneshwari V. (2015). Molecular Docking of Compounds Elucidated from Ixora Coccinea Linn. Flowers with Insulin Receptors. Scholars Researdh Libr. 7, 344–348.

[B17] Kathawala R. J. Gupta P. Ashby C. R. Chen Z. S. (2015). The Modulation of ABC Transporter-Mediated Multidrug Resistance in Cancer: A Review of the Past Decade. Drug Resist. Updat 18, 1–17. 10.1016/j.drup.2014.11.002 25554624

[B18] Kim Y. Chen J. (2018). Molecular Structure of Human P-Glycoprotein in the ATP-Bound, Outward-Facing Conformation. Science 359, 915–919. 10.1126/science.aar7389 29371429

[B19] Kwapisz D. (2017). Cyclin-dependent Kinase 4/6 Inhibitors in Breast Cancer: Palbociclib, Ribociclib, and Abemaciclib. Breast Cancer Res. Treat. 166, 41–54. 10.1007/s10549-017-4385-3 28741274

[B20] Lee T. D. Lee O. W. Brimacombe K. R. Chen L. Guha R. Lusvarghi S. (2019). A High-Throughput Screen of a Library of Therapeutics Identifies Cytotoxic Substrates of P-Glycoprotein. Mol. Pharmacol. 96, 629–640. 10.1124/mol.119.115964 31515284 PMC6790066

[B21] Li Q. Liu X. Zhang M. Ye G. Qiao Q. Ling Y. (2010). Characterization of A Novel Human CDK5 Splicing Variant that Inhibits Wnt/Beta-Catenin Signaling. Mol. Biol. Rep. 37, 2415–2421. 10.1007/s11033-009-9752-7 19693690

[B22] Li M. Yin D. Li J. Shao F. Zhang Q. Jiang Q. (2020). Rosmarinic Acid, the Active Component of Salvia Miltiorrhizae, Improves Gliquidone Transport by Regulating the Expression and Function of P-Gp and BCRP in Caco-2 Cells. Pharmazie 75, 18–22. 10.1691/ph.2020.9754 32033628

[B23] Liang S. Hu L. Wu Z. Chen Z. Liu S. Xu X. (2020). CDK12: A Potent Target and Biomarker for Human Cancer Therapy. Cells 9, 1483. 10.3390/cells9061483 PMC7349380 32570740

[B24] Liu Z. Long X. Chao C. Yan C. Wu Q. Hua S. (2014). Knocking Down CDK4 Mediates the Elevation of Let-7c Suppressing Cell Growth in Nasopharyngeal Carcinoma. BMC Cancer 14, 274. 10.1186/1471-2407-14-274 24751144 PMC4014407

[B25] Loyer P. Trembley J. H. (2020). Roles of CDK/Cyclin Complexes in Transcription and Pre-mrna Splicing: Cyclins L and CDK11 at the Cross-Roads of Cell Cycle and Regulation of Gene Expression. Semin. Cel Dev Biol 107, 36–45. 10.1016/j.semcdb.2020.04.016 32446654

[B26] Martínez-Chávez A. van Hoppe S. Rosing H. Lebre M. C. Tibben M. Beijnen J. H. (2019). P-glycoprotein Limits Ribociclib Brain Exposure and CYP3A4 Restricts its Oral Bioavailability. Mol. Pharm. 16, 3842–3852. 10.1021/acs.molpharmaceut.9b00475 31329454

[B27] Menzl I. Zhang T. Berger-Becvar A. Grausenburger R. Heller G. Prchal-Murphy M. (2019). A Kinase-independent Role for CDK8 in BCR-Abl1+ Leukemia. Nat. Commun. 10, 4741. 10.1038/s41467-019-12656-x 31628323 PMC6802219

[B28] Mi Y. J. Liang Y. J. Huang H. B. Zhao H. Y. Wu C. P. Wang F. (2010). Apatinib (YN968D1) Reverses Multidrug Resistance by Inhibiting the Efflux Function of Multiple ATP-Binding Cassette Transporters. Cancer Res. 70, 7981–7991. 10.1158/0008-5472.CAN-10-0111 20876799 PMC2969180

[B29] Rainey M. D. Quachthithu H. Gaboriau D. Santocanale C. (2017). DNA Replication Dynamics and Cellular Responses to ATP Competitive CDC7 Kinase Inhibitors. ACS Chem. Biol. 12, 1893–1902. 10.1021/acschembio.7b00117 28560864

[B30] Rank K. B. Evans D. B. Sharma S. K. (2000). The N-Terminal Domains of Cyclin-dependent Kinase Inhibitory Proteins Block the Phosphorylation of Cdk2/Cyclin E by the CDK-Activating Kinase. Biochem. Biophys. Res. Commun. 271, 469–473. 10.1006/bbrc.2000.2648 10799321

[B31] Robert T. Johnson J. L. Guichaoua R. Yaron T. M. Bach S. Cantley L. C. (2020). Development of A CDK10/CycM *In Vitro* Kinase Screening Assay and Identification of First Small-Molecule Inhibitors. Front. Chem. 8, 147. 10.3389/fchem.2020.00147 32175313 PMC7056863

[B32] Sorf A. Sucha S. Morell A. Novotna E. Staud F. Zavrelova A. (2020). Targeting Pharmacokinetic Drug Resistance in Acute Myeloid Leukemia Cells with CDK4/6 Inhibitors. Cancers (Basel) 12, 1596. 10.3390/cancers12061596 PMC7352292 32560251

[B33] Szwarcwort-Cohen M. Kasulin-Boneh Z. Sagee S. Kassir Y. (2009). Human Cdk2 Is A Functional Homolog of Budding Yeast Ime2, the Meiosis-specific Cdk-like Kinase. Cell Cycle 8, 647–654. 10.4161/cc.8.4.7843 19197163

[B34] Tai T. S. Lin P. M. Wu C. F. Hung S. K. Huang C. I. Wang C. C. (2019). CDK4/6 Inhibitor LEE011 Is a Potential Radiation-Sensitizer in Head and Neck Squamous Cell Carcinoma: An *In Vitro* Study. Anticancer Res. 39, 713–720. 10.21873/anticanres.13167 30711949

[B35] Tripathy D. Bardia A. Sellers W. R. (2017). Ribociclib (LEE011): Mechanism of Action and Clinical Impact of This Selective Cyclin-dependent Kinase 4/6 Inhibitor in Various Solid Tumors. Clin. Cancer Res. 23, 3251–3262. 10.1158/1078-0432.CCR-16-3157 28351928 PMC5727901

[B36] Wu T. Chen Z. To K. K. W. Fang X. Wang F. Cheng B. (2017). Effect of Abemaciclib (LY2835219) on Enhancement of Chemotherapeutic Agents in ABCB1 and ABCG2 Overexpressing Cells *In Vitro* and *In Vivo* . Biochem. Pharmacol. 124, 29–42. 10.1016/j.bcp.2016.10.015 27816545

[B37] Wu C. P. Hung T. H. Hsiao S. H. Huang Y. H. Hung L. C. Yu Y. J. (2020). Erdafitinib Resensitizes ABCB1-Overexpressing Multidrug-Resistant Cancer Cells to Cytotoxic Anticancer Drugs. Cancers (Basel) 12, 1366. 10.3390/cancers12061366 PMC7352346 32466597

[B38] Yang D. Kathawala R. J. Chufan E. E. Patel A. Ambudkar S. V. Chen Z. S. (2014). Tivozanib Reverses Multidrug Resistance Mediated by ABCB1 (P-Glycoprotein) and ABCG2 (BCRP). Future Oncol. 10, 1827–1841. 10.2217/Fon.13.253 24295377 PMC8366683

[B39] Yoshimura A. Kuwazuru Y. Sumizawa T. Ikeda S. Ichikawa M. Usagawa T. (1989). Biosynthesis, Processing and Half-Life of P-Glycoprotein in A Human Multidrug-Resistant KB Cell. Biochim. Biophys. Acta 992, 307–314. 10.1016/0304-4165(89)90089-5 2570611

[B40] Zhang L. Li Y. Wang Q. Chen Z. Li X. Wu Z. (2020). The PI3K Subunits, P110α and P110β Are Potential Targets for Overcoming P-Gp and BCRP-Mediated MDR in Cancer. Mol. Cancer 19, 10. 10.1186/s12943-019-1112-1 31952518 PMC6966863

[B41] Zhang L. Li Y. Hu C. Chen Y. Chen Z. Chen Z.-S. (2022). CDK6-PI3K Signaling axis Is an Efficient Target for Attenuating ABCB1/P-Gp Mediated Multi-Drug Resistance (MDR) in Cancer Cells. Mol. Cancer. In press. 10.1186/s12943-022-01524-w PMC9027122 35459184

