# Supplementary material for: Ribociclib Inhibits P-gp-Mediated Multidrug Resistance in Human Epidermoid Carcinoma Cells
Source: Front Pharmacol. 2022 Apr 1;13:867128. doi: 10.3389/fphar.2022.867128 (PMC9016416; doi:10.3389/fphar.2022.867128)
Supplement: Supplementary file 1 [file DataSheet1.docx]

**Supporting materials**


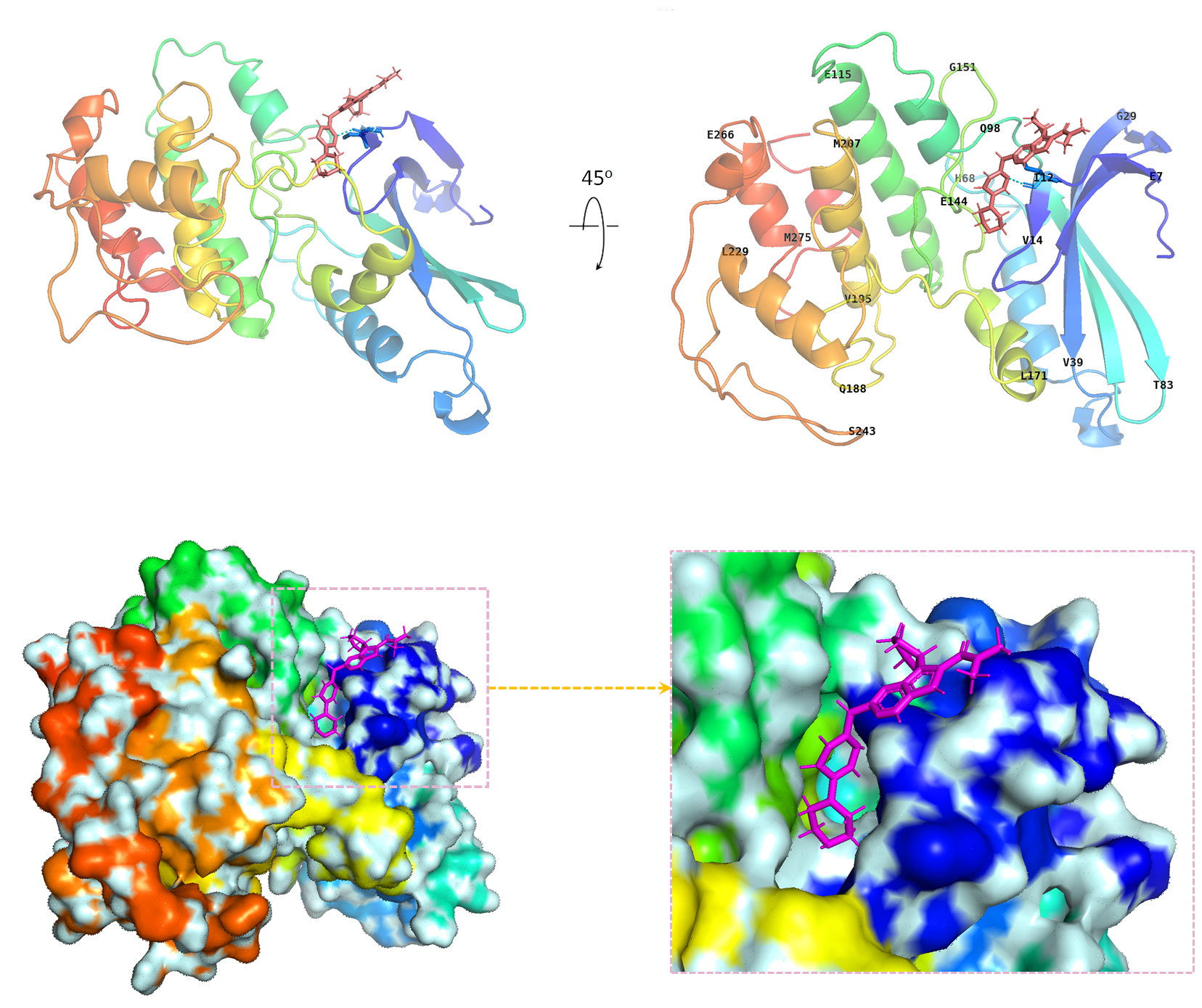


**FIGURE S1⏐**Docking analysis showing one of the potential binding sites for CDK4 to receive inhibitor ribociclib. Ribociclib shows good affinity to CDK4 at the binding pocket between the N-terminal and C-terminal domain.


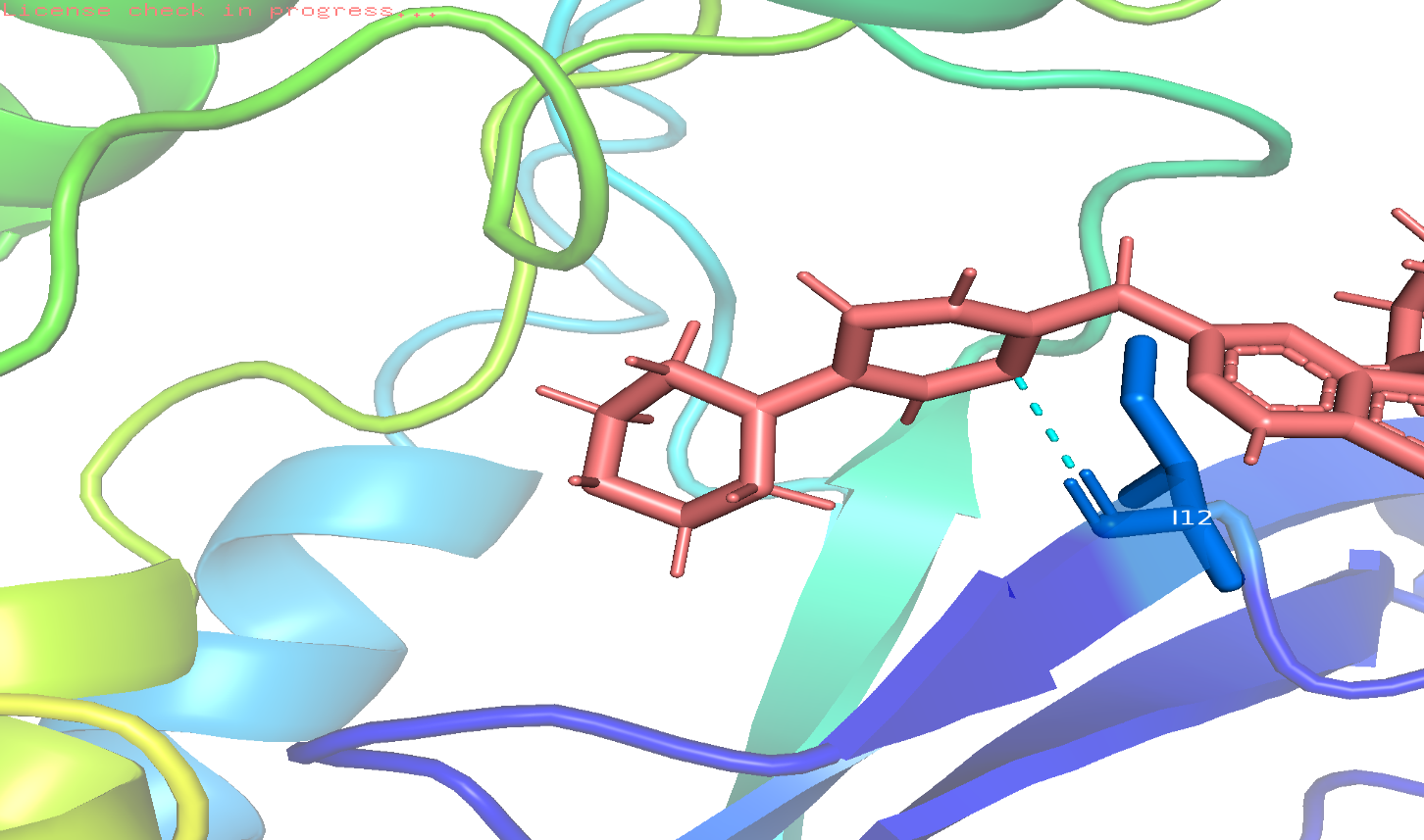


**FIGURE S2⏐**Docking analysis showing the structure of the CDK4-ribociclib complex. Polar interaction was detected between ribociclib and I12 of CDK4.


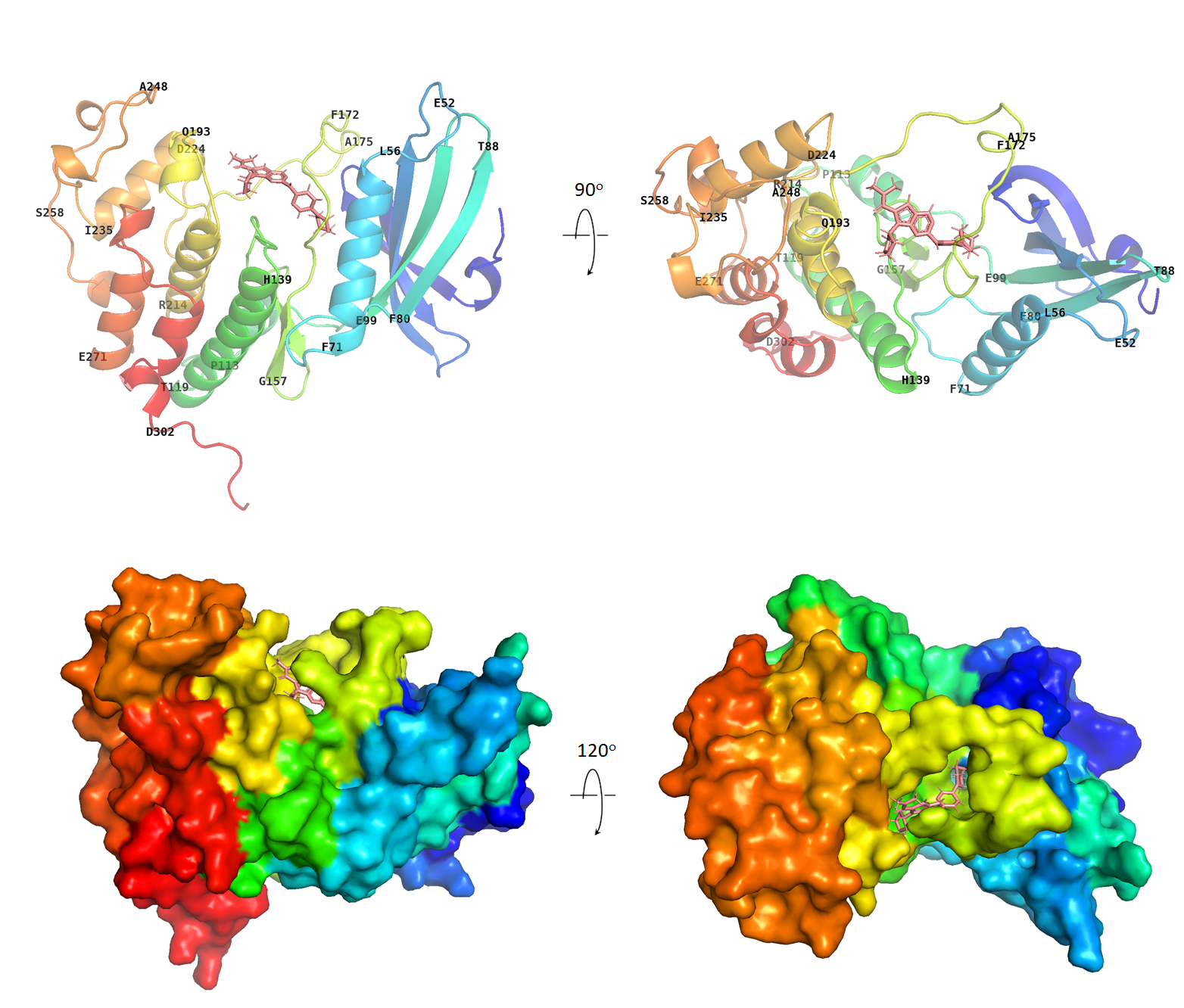


**FIGURE S3⏐**Docking analysis showing one of the potential binding sites on CDK6 for ribociclib. Ribociclib had significant interaction with the cave between the N’-and C’-terminal domains.

**
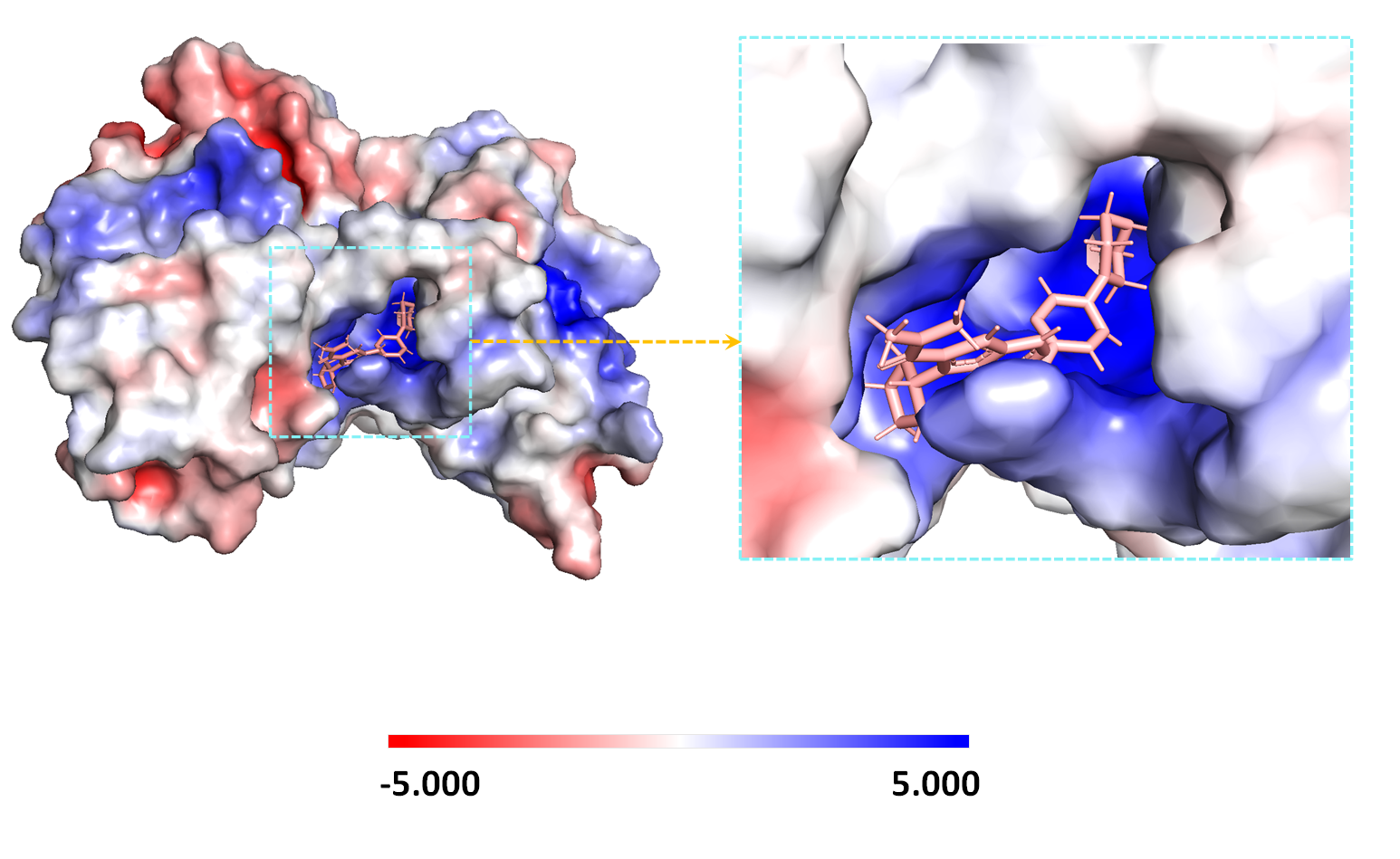
**

**FIGURE S4⏐**One of the docking models showing the spatial distribution of the charges on the surface of CDK6 for ribociclib. In addition to structure matching, the cave where ribociclib fits have positive charges, which can attract the solitary electron pairs provided by the nitrogen atom in the heterocyclic structures.
